# Modeling Approach for Reactive Injection Molding of Polydisperse Suspensions with Recycled Thermoset Composites

**DOI:** 10.3390/polym16162245

**Published:** 2024-08-07

**Authors:** Bhimesh Jetty, Florian Wittemann, Luise Kärger

**Affiliations:** Department of Lightweight Engineering, Karlsruhe Institute of Technology (KIT), Rintheimer-Querallee 2, 76131 Karlsruhe, Germany

**Keywords:** reactive injection molding, highly filled thermoset suspension, recycled thermoset composite, multiphase flow, OpenFOAM

## Abstract

Recycling production waste in the reactive injection molding (RIM) process is a step towards sustainability and efficient material usage. The recycled thermoset composite (RTC) material obtained by shredding the production waste is reused with a virgin thermoset composite (VTC). This study presents a mold-filling simulation approach considering this polydisperse suspension of RTC and VTC. Mold-filling simulations can assist in predicting processability and assessing the impact of reinforced RTC on the final part of production. State-of-the-art mold-filling simulations use the Cross–Castro–Macosko (CCM) model or anisotropic fiber-orientation-dependent viscosity models. The rheological parameters are determined either for the VTC or neat resin. However, these models do not account for changes in viscosity due to the reinforcing of fillers such as RTC. An effective viscosity model is developed by extending the CCM model using the stress–strain amplification approach to overcome this gap. This model is implemented in the computational fluid dynamics code OpenFOAM, and simulations are performed using an extended multiphase solver. To validate the simulations, experimental trials were executed using a two-cavity mold equipped with pressure sensors. Molding compounds with different compositions of VTC and RTC were injected at different speeds. Reinforcing VTC with RTC increases the viscosity. Results demonstrate that RTC-reinforced compounds require higher injection pressure for mold filling than VTC alone. The qualitative agreement of pressure profiles from simulations and experiments for different proportions of reinforcing RTC and different injection speeds shows that the implemented viscosity model can reproduce the experimental mold-filling behavior.

## 1. Introduction

Reinforced thermoset composites consist of a thermoset resin reinforced with discontinuous fillers in fibrous or particulate form (such as fibers and minerals) in a predetermined composition. The combined properties of the resin and fillers enhance the strength and stiffness and aid in weight reduction in the final fabricated part. Because of this characteristic, thermoset composites are used to manufacture intricate lightweight components in the automotive and aerospace industries. The manufacturing technique for thermoset composites using injection molding is also called reactive injection molding (RIM) [1]. In the RIM process, the B-stage thermoset molding compound is in the form of granules or pellets. These are melted into a liquid state in the plasticizing unit of the injection molding machine. Subsequently, this resin melt is injected under high pressure (up to 270 MPa during the mold-filling phase in the present study) into a mold cavity maintained at a temperature higher than that of the melt. As the melt is exposed to heat, it undergoes an irreversible exothermic reaction known as curing. This curing process results in the chemical cross-linking of polymer chains, causing the melt to solidify [2,3]. In contrast to thermoplastics, cured thermoset parts cannot be remolded because of their cross-linked structure and present a substantial challenge for recycling [4].

Over the past three decades, research has been conducted to investigate various methods, such as thermal, chemical, and mechanical recycling, to address the challenge of recycling thermoset composites. Each recycling method has its limitations, such as incomplete recycling, high costs, and potential degradation of material properties. Thermal recycling involves decomposing the polymer matrix at high temperatures, whereas chemical recycling breaks down the polymer chains into monomers or other chemicals [5,6,7,8,9]. Nevertheless, achieving complete recycling of thermoset plastic parts remains a major challenge [10]. This study focuses on the processability of a mechanically recycled thermoset composite. Specifically, the production waste of the RIM process itself, the sprue and runner system, is shredded into fine powder or granules. The resulting recycled thermoset composite (RTC) is reintroduced directly into the RIM process as an additional reinforcing material [5]. Within the framework of this study, the influence of mechanically recycled particles on mold-filling behavior is investigated. Reusing such recycled granular materials in subsequent mold filling is important for more sustainable material use. However, to realize satisfactory production, adequate process simulation is required to predict the impact of the recycled material on the process and the final part.

Such cured recycled particles are rigid and nearly spherical in shape. In addition, the matrix-to-filler ratio in the RTC is the same as that of the virgin thermoset composite (VTC). Here, VTC refers to the state of the material that is new and unprocessed previously. When the VTC, which includes prefilled fibrous or particulate fillers, is additionally reinforced with RTC, it results in a polydisperse suspension upon melting. This polydisperse suspension encompasses fillers of diverse shapes and sizes. The reinforcement leads to an effective increase in the total filler content of the molding compound. During the mold-filling process, this change in filler content imposes additional hindrance on the flow field at the microscopic level. As a result, the microscopic shear deformation substantially exceeds the applied macroscopic shear deformation. Depending on the extent of the increase in filler content, this elevated microscopic shear deformation translates into an increase in the bulk stress of the suspension at the macroscopic level [11].

RIM is a complex process that is affected by many factors, including material properties, mold design, and processing conditions. Therefore, simulation software is used to predict the outcome of the molding process and identify potential risks. Rheological modeling in thermoset injection molding is of utmost importance because the flow behavior of the material plays a critical role in shaping the overall molding process. For most state-of-the-art mold-filling simulations of the RIM process, the Cross–Castro–Macosko (CCM) model is employed. It describes the reactive viscosity of shear-thinning thermoset composites as a function of temperature, shear rate, and degree of cure [12,13,14,15,16,17,18]. Nonetheless, the CCM model does not account for the increased viscosity and altered flow characteristics introduced by reinforcing fillers such as RTC. In the case of fiber reinforcement, more comprehensive approaches involving a fourth-order anisotropic viscosity tensor can be applied. These advanced approaches consider additional factors such as fiber orientation, length, and volume fraction, providing a more detailed representation of the material’s flow behavior [14,19,20,21]. For the modeling of polydisperse suspensions, various approaches have been presented, e.g., for rigid spherical fillers of different sizes suspended in Newtonian fluid [22,23,24,25,26,27,28,29,30], and for a mixture of fillers containing rigid cylindrical and spherical fillers suspended in Newtonian fluid [31,32]. However, polydisperse suspensions with recycled particles and non-Newtonian thermoset resins have not yet been addressed. In this context, two approaches can be distinguished. One approach is to characterize the homogeneous material system comprising thermoset resin, fillers, and recycled particles, which is cost-intensive. Alternatively, if the material properties of the VTC are known, the previously mentioned rheological models can be extended to account for the effects of polydispersity. The latter approach is illustrated in this study, introducing a novel method to address this research gap.

The objective of this work is not just to simulate the mold-filling of a thermoset compound reinforced with recycled granules but to contribute to the broader goal of sustainable manufacturing. In this context, a novel viscosity model is proposed by extending the CCM model using a phenomenological approach called the stress–strain amplification method [11,19,33,34,35]. The resulting constitutive model describes the reactive viscosity of the polydisperse suspension as a function of reinforcing filler content, temperature, shear rate, and degree of cure. This model is implemented in the finite volume simulation software OpenFOAM 6. Subsequently, mold-filling simulations are conducted and compared with experimental results for different weight fractions of the recycled composite under different injection speeds. By validating these simulations against experimental trials, this research provides valuable insights into the processability of recycled materials, a crucial step in the journey toward sustainable manufacturing.

## 2. Experimental Setup

The experimental trials aimed to investigate the influence of recycled content on mold-filling behavior. All the experimental trials were conducted in collaboration with Fraunhofer ICT, based in Pfinztal, Germany. A commercially available, highly filled thermoset phenolic molding compound was used in these experimental trials. SBHPP Vyncolit, based in Gent, Belgium, manufactured and supplied the molding compound. It contains fillers, including glass fibers, silicon dioxide, and wollastonite, with weight fractions of 15%, 25%, and 40%, respectively. Therefore, the total weight fraction of the fillers $\phi_{{Tot}_{w}}$ in the molding compound is 80%.

**Figure 1:**
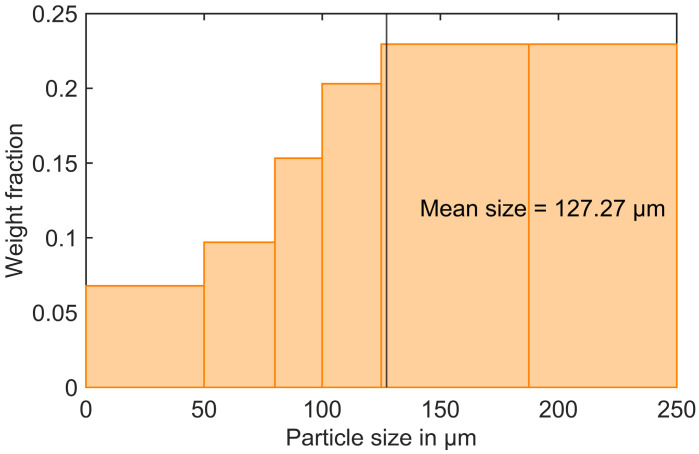
The particle size distribution of the RTC particles was obtained using sieve analysis.

**Figure 2:**
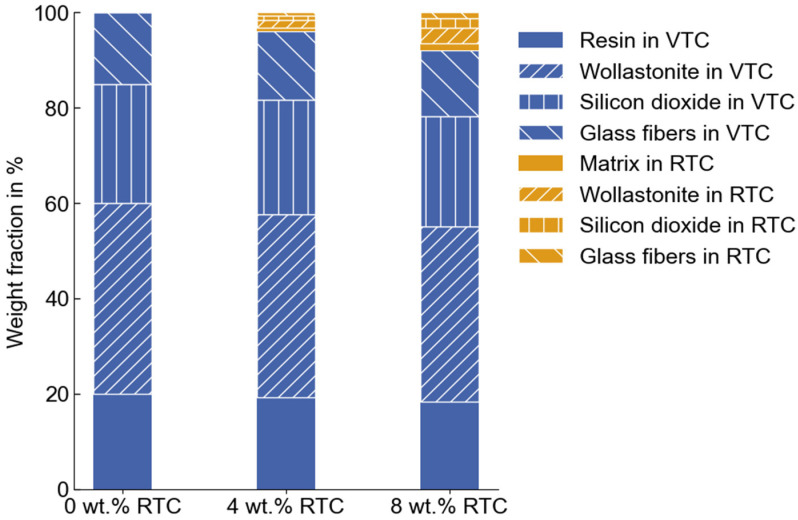
Composition of fillers in the molding compound for different proportions of reinforced RTC.

The production waste of the molded component is shredded into fine powders or granules. Particles with a diameter of less than 250 μm are separated using sieve analysis. The resulting particle size distribution is shown in Figure 1. With varying weight fractions of 4% and 8%, the VTC is reinforced with the RTC. The corresponding composition of fillers before and after reinforcement of the recycled particle content is presented in Figure 2.

**Figure 3:**
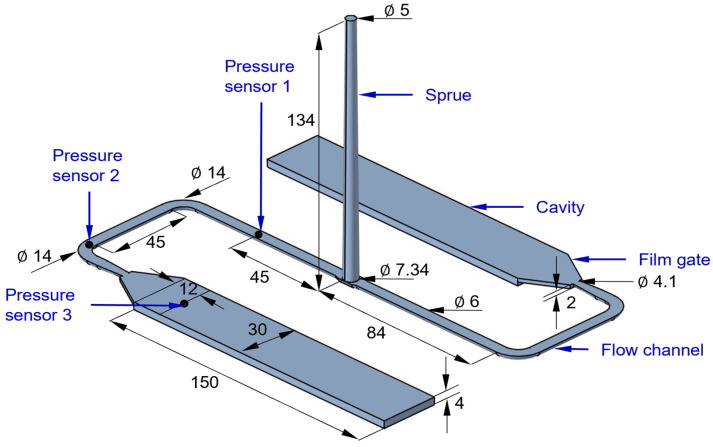
Symmetric two-cavity mold equipped with pressure sensors. All dimensions are in mm.

The injection molding experiments were conducted using an Arburg 320C 600–250 injection molding machine, which features a three-zone plasticizing unit with a 30 mm diameter screw. This machine supports a maximum injection pressure of 247 MPa and is equipped with an electrically heated two-plate injection molding tool. The mold cavity considered in the experiments is shown in Figure 3. It consists of a sprue designed as the frustum of a cone, extending into two identical semicircular flow channels. These flow channels are connected to the cavities through a film gate. To study the mold-filling behavior, three pressure sensors of type 6163A, manufactured by the Kistler Group (based in Winterthur, Switzerland), were placed along the flow path to monitor the development of the internal pressure during the mold-filling phase. Two sensors are positioned at the beginning and end of the flow channel, and the third sensor is placed at the beginning of the cavity.

**Figure 4:**
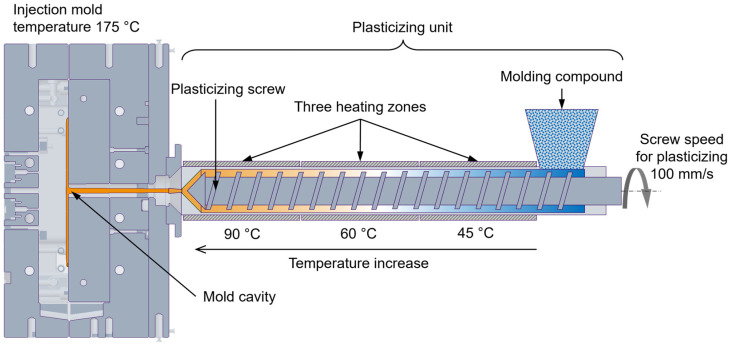
Processing conditions in the RIM [16].

First, injection molding experiments using VTC are performed. The processing conditions employed are illustrated in Figure 4. The temperature profile of the three-zone plasticizing unit is set to 45–60–90 °C. The plasticizing volume and switchover point V/P were set to 63 cm^3^ and 7 cm^3^, respectively. The screw speed for plasticizing and back pressure was set to 100 mm/s and 70 bar, respectively. These experiments used injection speeds of 10 cm^3^/s and 15 cm^3^/s. Five injection cycles were performed for each set of processing conditions to ensure the reproducibility of the experimental results against process-induced uncertainties. Subsequently, trials were conducted using VTC reinforced with 4% and 8% RTC. These trials followed a procedure identical to those using the VTC. The mean internal cavity pressure profiles are determined for each configuration of the processing conditions. These experimental pressure profiles are used in Section 5.2 to validate the simulation of the mold-filling behavior, ensuring accuracy and reliability in the analysis.

## 3. Process and Material Modeling

In this work, the mold-filling simulation of the RIM process is modeled using the numerical method of computational fluid dynamics. For this purpose, the standard volume of fluid (VoF)-based multiphase solver compressibleInterFoam in OpenFOAM 6 was extended. This section describes the governing equations and constitutive models.

### 3.1. Governing Equations

In FVM, the computational domain is discretized into a finite number of individual cells known as control volumes. The governing equations are solved for each of the control volumes. The conservation of mass is represented by the following compressible form of the continuity equation:

(1) $$\frac{\partial \rho }{\partial t}+\nabla \cdot \left(\rho v\right)=0,$$

where ρ is the mass density, t is the time, and v is the velocity vector. The first term on the left-hand side represents the rate of change in density with respect to time in the control volume. The second term on the left-hand side represents mass flow across the boundary faces of the control volume. The conservation of momentum for the compressible fluid is given by the following equation:

(2) $$\frac{\partial (\rho v)}{\partial t}+\nabla \cdot \left((\rho v)⨂v\right)=-\nabla p+\nabla \cdot \tau +\rho g,$$

where p is the pressure, τ is the viscous stress tensor, and g is the gravity vector. On the left-hand side of the momentum equation, the first term represents the rate of change in momentum with respect to time in the control volume. The second term represents the flow of momentum in and out across the control volume. On the right-hand side of the momentum equation, the first and second terms represent hydrostatic pressure and shear stresses acting on the control volume. The third term on the right-hand side accounts for the effect of gravity. For a Newtonian fluid, τ is given by the following equation:

(3) $$\tau =\;2\eta D-\frac{2}{3}\eta \delta 𝛻\cdot v,$$

where η is the scalar viscosity of the fluid, D is the strain rate tensor, and δ is the second-order identity tensor. The deformation rate tensor is given by the following equation:

(4) $$D=\frac{1}{2}\left[\nabla v+{\left(\nabla v\right)}^{T}\right].$$

The temperature field is evaluated using the following equation:

(5) $$\frac{\partial \left(\rho c_{p}T\right)}{\partial t}+\nabla \cdot \left(\left(\rho c_{p}T\right)\cdot v\right)=\nabla \;\cdot (\lambda \;\nabla T)+\tau :D+\dot{Q},$$

where T is the temperature, $c_{p}$ is the specific heat capacity at constant pressure, and λ is the thermal conductivity. On the left-hand side, the first term represents the rate of change in enthalpy with respect to time. The second term represents the convective transport of enthalpy due to the motion of the fluid. On the right-hand side of the energy equation, the first term represents the diffusion of thermal energy through the fluid due to temperature gradient. Moreover, τ:D accounts for the viscous heat dissipation, while $\dot{Q}=\rho H_{r}\dot{c}$ represents the heat source resulting from the exothermic reaction of the curing thermoset. Here, $\dot{c}$ is the rate of cure calculated from the reaction kinetics model (see Equation (26) in Section 3.2.2), and $H_{r}$ is the reaction enthalpy of the thermoset. The partial differential terms in the three governing equations are solved using a first-order accurate upwind scheme.

In addition to the governing equations, the phase fraction equation is solved. In the RIM process, the mold is initially occupied by air. The thermoset melt is then injected into the mold. To distinguish between these two fluid phases during mold filling, a scalar quantity $\alpha \;\in \left[0,1\right]$ is used to evaluate the phase fraction. If the phase fraction is α=1, the cell volume is entirely occupied by the thermoset compound. The phase fraction α=0 implies that the cell volume is occupied by air. For other values of α, the cell volume is shared between the polymer and air. The phase fraction is evaluated using the volume of fluid approach. According to the volume of fluid approach, the phase fraction is evaluated using the following equation:

(6) $$\frac{\partial \alpha }{\partial t}+\nabla \cdot \left(\alpha v\right)+\nabla \cdot \left[\alpha \left(1-\alpha \right)v_{r}\right]=S,$$

where $v_{r}$ is the measure of relative velocity between two fluid phases. It is also called compression velocity. In this study, $v_{r}$ is given as follows:

(7) $$v_{r}=v_{resin}-v_{air},$$

where $v_{resin}$ and $v_{air}$ are the velocities of resin and air. S represents the source term arising from the influence of pressure on the densities and specific compressibility of both fluid phases. The detailed definition of this source term can be found in [36].

### 3.2. Constitutive Material Models

#### 3.2.1. Extension of the Reactive Viscosity Model for Polydisperse Suspensions

The following introduces the underlying assumptions for the effective viscosity of polydisperse thermoset suspensions. This study assumes that the fillers in the VTC and RTC particles are unbreakable and retain their original size under any applied macroscopic shear deformation. The attrition of fillers during compounding, plasticizing, and mold filling leads to their size reduction. Due to process-induced fluctuations, this reduction varies from one injection cycle to the next, even under identical processing conditions. Thereby influencing viscosity and consequently affects the accuracy of flow modeling. While flow modeling with fiber breakage in fiber-reinforced thermoset polymers has been successfully demonstrated [37], flow modeling considering the attrition of fillers in a polydisperse suspension is complex and is yet to be addressed. Especially when considering multiparticle interactions and forces acting on these fillers. To circumvent this complexity, the assumption as mentioned above is made to simplify the modeling of viscosity. The fillers of each shape class are of uniform size.

Due to the lack of specific data on the thermal properties of RTC, it is assumed that there is no significant difference in the thermal properties of the composite across varying degrees of curing. This assumption is based on the high filler content in the molding compound, comprising 80 wt.% of fillers, which dominates the thermal properties. The Recycled Thermoset Composite (RTC) is the cured form of the Virgin Thermoset Composite (VTC). During processing with RTC, only 4 to 8 wt.% are reinforced, which does not significantly influence the thermal properties of the molding compound comprising VTC and RTC. This assumption is consistent with the mold-filling simulations of thermoset injection molding in the literature, where thermal properties are typically considered independent of the degree of cure [15].

When we let $V_{VTC}$ be the total volume of fillers in VTC, it is expressed as follows:

(8) $$V_{VTC}=V_{GF}+V_{Sil}+V_{Wol},$$

where $V_{GF}$, $V_{Sil}$, and $V_{Wol}$ represent the volume of glass fibers, silicon dioxide, and wollastonite, respectively. Let $V_{Rcy}$ be the volume of the RTC. When the recycled particles of volume $V_{Rcy}$ are added to VTC, the filler volume fractions $\phi_{{VTC}_{v}}$ from virgin material in the total suspension can be calculated using the following equation:

(9) $$\phi_{{VTC}_{v}}=\frac{V_{VTC}}{V_{Mat}+V_{VTC}+V_{Rcy}},$$

where $V_{Mat}$ is the volume of the thermoset matrix corresponding to the uncured state. The filler volume fraction of recycled particles $\phi_{{Rcy}_{v}}$ is given by the following equation:

(10) $$\phi_{{Rcy}_{v}}=\frac{V_{Rcy}}{V_{Mat}+V_{VTC}+V_{Rcy}},$$

From Equations (9) and (10), the total volume fraction of the fillers in the polydisperse suspension can be expressed as follows:

(11) $$\phi_{{Tot}_{v}}=\phi_{{VTC}_{v}}+\phi_{{Rcy}_{v}}.$$

Let $\phi_{{VTC}_{v}}^{*}$ be the volume fraction of fillers in VTC before reinforcement with RTC (at $V_{Rcy}=0$, in Equation (9), which is given by the following equation:

(12) $$\phi_{{VTC}_{v}}^{*}=\frac{V_{VTC}}{V_{Mat}+V_{VTC}}.$$

The viscosity of the VTC can be evaluated using the Cross–Castro–Macosko (CCM) model, given by the following equation [38]:

(13) $$\eta_{\phi_{{VTC}_{v}}^{*}}=\frac{\eta_{0}\left(T\right)}{1+{\left(\frac{\eta_{0}\left(T\right)\;\dot{\gamma }}{\tau^{*}}\right)}^{\left(1-n\right)}}{\left(\frac{c_{g}}{c_{g}-c}\right)}^{\left(c_{1}+c_{2}\;c\right)},$$

with

(14) $$\eta_{0}\left(T\right)=B\;e^{\left(\frac{T_{b}}{T}\right)}.$$

In Equations (13) and (14), B, $T_{b}$ $\tau^{*}$, n, $c_{1}$, and $c_{2}$ are empirical material-specific parameters, and $c_{g}$ is the point of gelation. The parameters were determined for VTC through rheological characterization. $\dot{\gamma }$ is the scalar shear rate. Equation (13) can be extended using the phenomenological stress–strain amplification approach to evaluate the viscosity for the reinforcement of the VTC with RTC [11]. According to this approach, the effective viscosity of the resulting polydisperse suspension $\eta_{\phi_{{Tot}_{v}}}$ is expressed by the following equation:

(15) $$\eta_{\phi_{Totv}}=\eta_{\phi_{{VTC}_{v}|F_{\dot{\gamma }}}^{*}}\;\chi_{\phi_{{Rcy}_{v}}},$$

where $\eta_{\phi_{{VTC}_{v}|F_{\dot{\gamma }}}^{*}}$ is the viscosity of the suspending medium representing VTC, and $\chi_{\phi_{{Rcy}_{v}}}$ is the hydrodynamic amplification factor. The viscosity $\eta_{\phi_{{VTC}_{v}|F_{\dot{\gamma }}}^{*}}$ is the evaluation of $\eta_{\phi_{{VTC}_{v}}^{*}}$ incorporating the strain amplification factor $F_{\dot{\gamma }}$. Because the rheological parameters of the suspending medium are determined for VTC, the corresponding shear strain rate experienced over the suspending medium is amplified by a factor of ${\left(1-\phi_{{Rcy}_{v}}\right)}^{-1}$ due to the presence of a reinforcing fraction $\phi_{{Rcy}_{v}}$ of fillers in the suspension. The shear amplification factor $F_{\dot{\gamma }}$ for a suspension in a shear flow is given by the following equation [11]:

(16) $$F_{\dot{\gamma }}=\frac{1}{1-\phi_{{Rcy}_{v}}}.$$

Thus, $\eta_{\phi_{{VTC}_{v}|\dot{F_{\dot{\gamma }}}}^{*}}$ in Equation (15) yields

(17) $$\eta_{\phi_{{VTC}_{v}|\dot{F_{\dot{\gamma }}}}^{*}}=\frac{\eta_{0}\left(T\right)}{1+{\left(\frac{\eta_{0}\left(T\right)\;F_{\dot{\gamma }}\;\dot{\gamma }}{\tau^{*}}\right)}^{\left(1-n\right)}}\;{\left(\frac{c_{g}}{c_{g}-c}\right)}^{\left(c_{1}+c_{2}\;c\right)}.$$

The hydrodynamic amplification factor $\chi_{\phi_{{Rcy}_{v}}}$ in Equation (15) describes the amplification of shear viscosity due to the reinforcing fraction of particles $\phi_{{Rcy}_{v}}$ when the applied macroscopic shear rate is zero. Depending on the concentration of the fillers in the suspending medium and the concentration of the fillers to be added, there are two ways to model the hydrodynamic amplification factor. If the suspending medium is dilute or semi-dilute, it is modeled by the following equation [39,40]:

(18) $$\chi_{\phi_{{Rcy}_{v}}}=1+{\left[\eta \right]}_{1}\;\phi_{{Rcy}_{v}}+{\left[\eta \right]}_{2}\;\phi_{{Rcy}_{v}}^{2}+\cdots +{\left[\eta \right]}_{n}\;\phi_{{Rcy}_{v}}^{n},$$

where ${\left[\eta \right]}_{n}$ represents the coefficients of the polynomial expansion. The coefficient ${\left[\eta \right]}_{1}$ represents the intrinsic viscosity. It defines the increase in viscosity due to a single particle. The coefficient ${\left[\eta \right]}_{2}$ represents the second-order intrinsic viscosity. It represents the influence of the interaction of particle pairs on viscosity. Similarly, the higher-order coefficients represent the intrinsic viscosity due to multiparticle interactions. Thus far, the coefficients up to second-order terms have been analytically quantified. The higher-order coefficients are difficult to determine analytically considering multiparticle interactions. Therefore, higher-order terms are neglected [41].

When new fillers are added to a concentrated suspending medium, the effective viscosity of the suspension depends on the microstructure (or the geometrical arrangement of the fillers) in the suspension medium and the diffusion of all these fillers in the suspension under the application of a shear rate. The rate of diffusion of the fillers in the microstructure is the resulting effect of the forces acting on the particles. These are namely dispersive Brownian forces, hydrodynamic forces, and forces due to inter-particle interactions resulting from the exclusion of free volume (volume unoccupied by fillers) in the suspension. Furthermore, it is proportional to the free volume available for the suspended fillers in the suspended medium. Brownian forces are always present in the concentrated polydisperse suspensions, irrespective of the applied shear deformation. However, these forces are prominent for fillers with shapes on the order of a few nanometers to a few micrometers. Hence, as in this study, these forces are usually neglected [42].

The second way to model the hydrodynamic amplification factor $\chi_{\phi_{{Rcy}_{v}}}$ applies to highly concentrated suspensions. In such scenarios, further addition of small quantities of fillers can significantly influence the effective viscosity of the complete suspension, and thus, $\chi_{\phi_{{Rcy}_{v}}}$ is modeled using the Krieger and Dougherty equation [43]. This closed-form mathematical equation can also be expressed as a series of expansions with the objective of establishing a comparison to Equation (18) [23].

(19) $$\chi_{\phi_{{Rcy}_{v}}}=1+\left[\eta \right]\;\phi_{{Rcy}_{v}}+\frac{\left[\eta \right]}{2}\;\left(\left[\eta \right]+\frac{1}{\phi_{max}}\right)\;\phi_{{Rcy}_{v}}^{2}+\frac{\left[\eta \right]}{6}\;\left({\left[\eta \right]}^{2}+\frac{3[\eta ]}{\phi_{max}}+\frac{1}{\phi_{max}^{2}}\right)\;\phi_{{Rcy}_{v}}^{3}.$$

In Equation (19), the higher-order terms of $\phi_{{Rcy}_{v}}$ are neglected. $\left[\eta \right]$ is the intrinsic viscosity. Its value depends upon the shape (aspect ratio), orientation, and on the applied shear deformation of the filler in the suspending medium. In this study, the recycled particles are nearly spherical in shape. Unlike anisotropic fillers such as fibers, spherical filler particles do not exhibit orientation when the suspension is subjected to motion at a shear rate [44]. For spherical particle $\left[\eta \right]$ = 2.5 [39].

In Equation (19), $\phi_{max}$ is the geometrical maximum packing fraction of fillers in the total suspension. The inverse of the maximum packing fraction accounts for the effect of interparticle interactions due to the geometrical arrangement of fillers in the microstructure resulting from the exclusion of free volume in the suspension. For randomly packed mono-sized fillers, the maximum packing fraction can be determined by using the semi-empirical equation [45].

(20) $$\phi_{{max}_{mono}}=\frac{\phi_{{max}_{spheres}}}{s_{p}}\;e^{\left(f_{s}\right)},$$

where $\phi_{{max}_{spheres}}=0.64$ is the maximum packing fraction of randomly packed spheres [45]. Equation (19) is applicable to fillers with an aspect ratio greater than or equal to unity. $s_{p}$ is the sphericity given by the following equation [45].

(21) $$s_{p}=\frac{2A_{r}+1}{3A_{r}^{\frac{2}{3}}},$$

where $A_{r}$ is the aspect ratio of the filler. Exponent $f_{s}$ is given by the following equation [45]:

(22) $$f_{s}=-0.1334\;{\left(s_{p}-1\right)}^{4}+0.9367{\left(s_{p}-1\right)}^{3}-2.1099{\left(s_{p}-1\right)}^{2}+0.8507\left(s_{p}-1\right).$$

Sudduth [46], as well as Dörr et al. [26], employed the theoretical model proposed by Furnas [47] to calculate the maximum packing fraction for polydisperse random packing of fillers. While Furnas originally devised a model for determining the theoretical maximum packing fraction of spherical fillers with varying sizes, the present study adapts the model to compute the polydisperse maximum packing fraction for different shapes based on their sphericity [45]. The calculation of the maximum packing fraction revolves around the concept of successive addition of fillers with decreasing sphericity into the available free volume within the suspension. Initially, fillers with the highest sphericity are introduced into the free volume, occupying the monodisperse maximum packing fraction $\phi_{{max}_{mono}}$. The remaining space is then allocated to subsequent fillers with progressively lower sphericity. The maximum packing fraction after *i*th addition can be given as follows [45]:

(23) $$\phi_{{max}_{i}}=\phi_{{max}_{i-1}}+\left(1-\phi_{{max}_{i-1}}\right)\phi_{{max}_{{mono}_{i}}}.$$

for i>1. If i=1, then $\phi_{{max}_{1}}=\phi_{{max}_{{mono}_{1}}}$. Based on the sphericity, $\phi_{{max}_{{mono}_{1}}}$, $\phi_{{max}_{{mono}_{2}}}$, $\phi_{{max}_{{mono}_{3}}}$, and $\phi_{{max}_{{mono}_{4}}}$ correspond to the maximum packing fraction of monodisperse wollastonite, glass fibers, RTC, and silicon dioxide, respectively. In the present work, the theoretical polydisperse maximum fraction evaluated based on Equation (23) is given by the following expression:

(24) $$\phi_{max}=\phi_{{max}_{4}}.$$

where $\phi_{max}$ from Equation (24) is used to evaluate the stress amplification factor given in Equation (19). The shape characteristics of these fillers and filler volume fractions to evaluate $\phi_{max}$ are listed in Table 1.

The details of the shape characteristics of fillers in VTC are provided by the material manufacturer. The shape characteristics of RTC are obtained from sieve analysis and are discussed in Section 2. Furthermore, substituting Equations (17) and (19) into Equation (15) gives the effective viscosity of the polydisperse suspension.

(25) $$\eta_{\phi_{{Tot}_{v}}}=\frac{\chi_{\phi_{{Rcy}_{v}}}\;\eta_{0}\left(T\right)}{1+{\left(\frac{\eta_{0}\left(T\right)\;F_{\dot{\gamma }}\;\dot{\gamma }}{\tau^{*}}\right)}^{\left(1-n\right)}}\;{\left(\frac{c_{g}}{c_{g}-c}\right)}^{\left(c_{1}+c_{2}\;c\right)}.$$

If $\phi_{{Rcy}_{v}}=0$, Equation (25) becomes reduced to the CCM model (Equation (13)).

#### 3.2.2. Reaction Kinetics Model

During the RIM process, the thermoset melt undergoes an irreversible exothermic chemical reaction upon applying heat. To model the curing kinetics of the thermoset matrix, the *n*th-order reaction kinetics approach from Kamal–Malkin is used [48], where the degree of curing c is defined by the following equation:

(26) $$\frac{dc}{dt}=\left(K_{1}+K_{2}c^{m}\right){\left(1-c\right)}^{n}.$$

The parameters $K_{1}$ and $K_{2}$ are determined by:

(27) $$K_{1}=A_{1}e^{\frac{E_{1}}{R_{g}\;T}},$$

and

(28) $$K_{2}=A_{2}\;e^{\frac{E_{2}}{R_{g}T}}.$$

where $A_{1}$, $A_{2}$, $E_{1}$, $E_{2}$, m, and n are material-specific empirical parameters determined by experimental characterization. $R_{g}$ is the universal gas constant. Furthermore, if the reaction enthalpy $H_{r}$ of VTC is determined, then the corresponding heat source term $\dot{Q}$ in Equation (5) at different propositions of RTC can be calculated by:

(29) $$\dot{Q}=\rho \;\left(1-\phi_{{Rcy}_{v}}\right)H_{r}.$$

## 4. Mold-Filling Simulation in OpenFOAM

**Figure 5:**
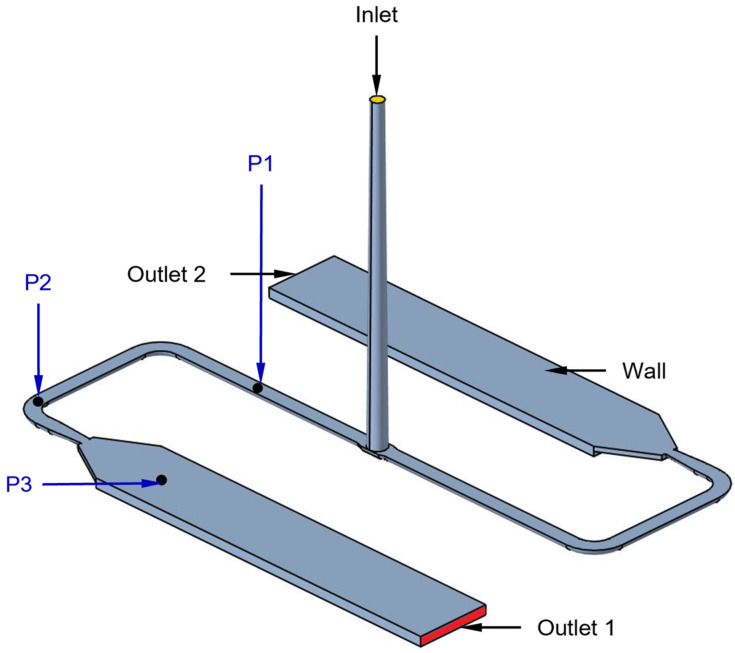
Boundary patches of the mold cavity with probing positions P1, P2, and P3 corresponding to the sensors are shown in Figure 3.

This section describes the mold-filling simulation of reactive injection molding in OpenFOAM based on the methodology described in Section 3. The mold cavity consists of four boundary patches: Inlet, Wall, Outlet 1, and Outlet 2, as shown in Figure 5. This mold cavity is initially occupied by air. During mold filling, the polymer melt enters the sprue from the inlet, flows through the runner, and enters the mold cavities through the film gates, while air exits through the outlet. The evolution of internal pressure during mold filling is monitored at positions P1, P2, and P3 in the simulation, which coincides with the sensor locations in Figure 3. The objective is to validate the simulated results for the given processing conditions and molding compounds by comparison with experimental results.

For the numerical simulation, the computational domain of the mold cavity is discretized into a finite number of control volumes using the meshing software HyperMesh 2021.2. A hexahedral mesh with an edge length of 0.5 mm along the flow direction is generated. In addition, elements with an edge length of 0.2857 mm were generated along the direction perpendicular to the flow, further enhancing the accuracy of the simulation. The generated mesh is then exported to OpenFOAM. The values of the field variables at each boundary patch and initial internal fluid domain are detailed in Table 2. The mold-filling simulation is carried out until the switchover fill volume is achieved, ensuring a comprehensive analysis.

The simulations are carried out using different propositions of VTC and RTC, as in the experimental trials. Depending on the filler volume fraction $\phi_{{Rcy}_{v}}$ of the recycled material, the reactive viscosity is evaluated using Equation (25). The material parameters of the VTC are given in Table 3. Furthermore, the parameters given in Table 4 are used to evaluate the curing kinetics. These material parameters are provided by the material manufacturer.

## 5. Results and Discussion

### 5.1. Numerical Verification

**Figure 6:**
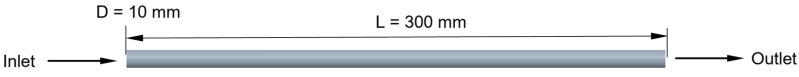
Cylindrical pipe and definition of boundary patches used for numerical verification.

Numerical verifications are performed to ensure accurate implementation of the viscosity model and illustrate the effect of reinforcing spherical fillers to Newtonian and non-Newtonian suspending medium. A 3D cylindrical pipe geometry as shown in Figure 6, measuring 300 mm in length and 10 mm in diameter, is used for verification. A hexahedral mesh with eight elements along the diameter and an edge length of 0.5 mm along the length of the pipe is generated.

The simulation setup is designed to replicate the Poiseuille flow of a Newtonian fluid. A pressure difference of 0.2 MPa is applied across the inlet and outlet boundary, and results are compared after attaining a steady state, i.e., after 50 s. A Newtonian fluid with a dynamic viscosity of 100 Pas, containing no suspended particles, is initially simulated. Subsequently, the impact of adding 20 vol.% mono-sized spherical fillers to this Newtonian suspending medium is analyzed by evaluating the viscosity and incorporating only the hydrodynamic amplification factor (as described in Equation (19)). Since Newtonian fluids do not exhibit shear-thinning behavior, the shear-thinning exponent is n=1. Therefore, the strain amplification factor, as described in Section 3.2.1 using Equation (16), is only relevant for non-Newtonian shear-thinning fluids and is not incorporated in the current case of a Newtonian Poiseuille flow. The effective viscosity is calculated only by considering the hydrodynamic amplification factor. For monomodal spheres, $\phi_{max}=\phi_{{max}_{spheres}}$. The resulting values of the hydrodynamic amplification factor and effective viscosity of the suspension are 1.78 and 178 Pas, respectively.

**Figure 7:**
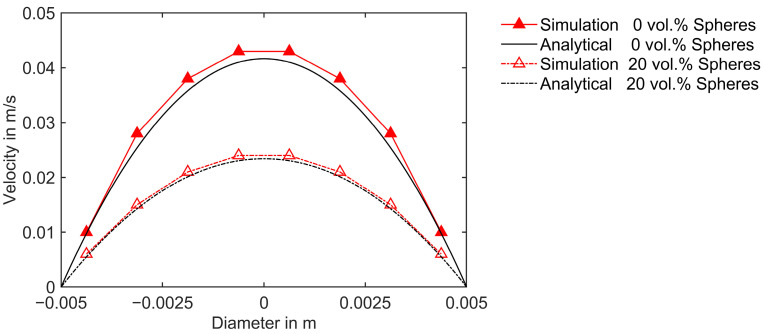
Comparison of numerical and analytical velocity profiles along the diameter for Newtonian fluid with 0 vol.% and 20 vol.% suspended spheres.

The simulation results are compared with analytical calculations. This comparison is crucial as it validates the accuracy of the model and the effectiveness of the numerical verification. The underlying equation for the analytical calculation of the velocity profile is described in [49]. The velocity profiles along the diameter of the pipe for the Newtonian fluid with and without spherical fillers are depicted in Figure 7. The good agreement between the simulation and the analytical velocity profiles indicates that the model is implemented correctly. It is observed from Figure 7 that the velocity profile of the fluid with the suspension of spheres lies below that of the fluid without the suspension of fillers. Due to hydrodynamic amplification, the resulting viscosity is higher for the fluid with suspensions compared to that without fillers. Consequently, under an identical applied pressure difference across the cylindrical pipe, the fluid with suspended spherical fillers flows with lower velocity.

The numerical verification is further extended to illustrate the effect of strain amplification for non-Newtonian shear-thinning fluids applying identical boundary conditions mentioned in the previous case. A numerical analysis of Poiseuille flow for a power-law fluid exhibiting shear-thinning behavior through cylindrical pipe geometry, as described in Figure 6, is conducted to achieve this objective. According to the power-law model, viscosity is defined as a function of shear rate according to the following expression [50]:

(30) $$\eta_{\dot{\gamma }}=m_{k}\;{\dot{\gamma }}^{n-1},$$

where $m_{k}$ is the consistency index, and n is the shear thinning exponent. This equation provides a simple approximation for calculating the viscosity of shear-thinning fluids. However, it carries a disadvantage when calculating viscosity at the limits of $\dot{\gamma }\to 0$ and $\dot{\gamma }\to \infty$. The hydrodynamic factor described in Section 3.2.1 using Equation (19) cannot be incorporated for the calculation of effective viscosity for the suspension of spherical fillers in power-law fluid. Here, the values for evaluating the power-law viscosity model, $m_{k}$ = 205 Pas^n^ and n = 0.7, are chosen. These values are chosen so that they yield a maximum velocity matching the previous case of the flow of Newtonian fluid.

First, simulations are executed using a power-law fluid without suspension of spheres. Subsequently, simulations with 20 vol.% mono-sized spherical fillers in this power-law shear thinning suspending medium are carried out by evaluating the effective viscosity incorporating the strain amplification factor, as described in Section 3.2.1 using Equation (16). After incorporating the strain amplification factor, Equation (30) can be rewritten as follows:

(31) $$\eta_{\dot{\gamma }|F_{\dot{\gamma }}}={\;m}_{k}\;(F_{\dot{\gamma }}\;{\dot{\gamma })}^{n-1}.$$

**Figure 8:**
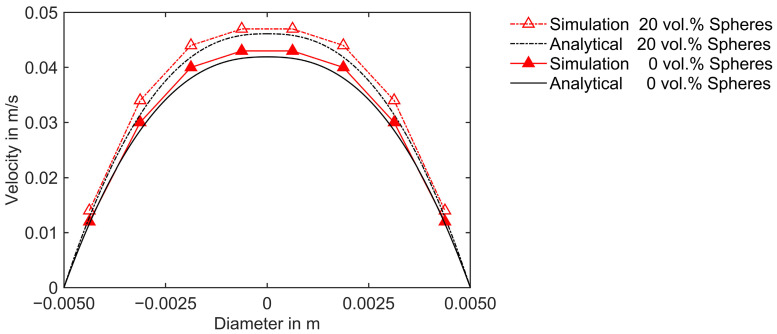
Comparison of numerical and analytical velocity profiles along the diameter for the power-law fluid with 0 vol.% and 20 vol.% suspended spheres.

The simulated velocity profiles in the direction perpendicular to the flow are compared with the analytical calculations in Figure 8. Similar to the previous case, the equations for the evaluation of the velocity profile are described in [49]. The results demonstrate a good agreement between the simulated and analytical velocity profiles, which indicates the model is implemented correctly. It is observed from Figure 8 that the velocity profile of the fluid with a suspension of spheres lies above that of the fluid without fillers. The reinforcement of fillers leads to an increase in strain amplification. This enhanced shearing decreases in viscosity for the shear-thinning fluid with spherical suspensions compared to that without fillers. Consequently, the fluid with suspended spherical fillers flows at a higher velocity under an identical pressure difference across the cylindrical pipe.

### 5.2. Validation of the Mold-Filling Simulations

**Figure 9:**
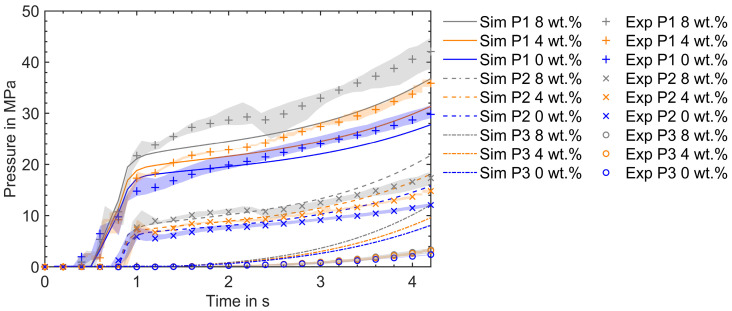
Pressure profiles during the filling phase at a flow rate of 10 cm^3^/s. The simulation and experimental results are plotted for different RTC proportions.

This section compares the simulation and experimental results in the mold-filling phase by varying the composition of the reinforced RTC and the injection speed. The following mold-filling simulation results are obtained by evaluating the viscosity model (Equations (16), (19), and (25)) described in Section 3.2.1 using material parameters described in Table 3 under Section 4. The pressure evolution in experimental trials over the mold-filling time is measured by pressure sensors along the flow path during an injection cycle under the processing conditions described in Section 2. One of the critical observations before comparing the pressure profiles is the difference in mold filling time in the experiment and simulation. Due to melt compression and backflow in the plasticizing unit, the mold filling time in the experimental trial was longer than in the simulation for the applied injection speed and processing conditions. Therefore, the experimental results are scaled to fit with the simulation results. In Figure 9, a comparison of the results with 0 wt.%, 4 wt.%, and 8 wt.% recycled content at a flow rate of 10 cm^3^/s is depicted. The experimental results are presented as the mean of five injection cycles and a min–max error band. This error band accounts for the process-induced fluctuations in the measured pressure values under the given processing conditions.

Reinforcing VTC with RTC increases viscosity. As a result, the molding compound reinforced with RTC requires a higher injection pressure than VTC. This distinction is visible in Figure 9, where the experimental pressure profiles corresponding to 8 wt.% and 4 wt.% reinforcement lie above those for 0 wt.% reinforcement of RTC, and 8 wt.% are higher than 4 wt.% RTC curves at sensor positions P1, P2, and P3, respectively. The offset between the pressure profiles is qualitatively reproduced by the simulations for different compositions of reinforcements of RTC. Nonetheless, there is a quantitative discrepancy in the pressure profiles between simulations and experiments at the sensor positions P1, P2, and P3.

At P1, the simulation underpredicts the pressure. Due to the reinforcement of RTC, the initial temperature and the initial degree of cure of the molding compound are significantly influenced. During plasticizing, the presence of additional fillers induces more shear heating. As a result, the initial melt temperature and the corresponding initial melt conversion are proportionately higher. In this study, identical values of temperature and initial degree of cure were applied at the inlet for all simulations, which may not be the case in experiments. A higher initial cure results in higher viscosity and temperature, and this discrepancy could be reflected in experiments where the measured pressure values at P1 are higher than the calculated values. Since there are no temperature sensors integrated into the injection mold around the probing point P1, the actual temperature measurements were not conducted in this study. This represents a limitation of this work, as direct validation of the simulated temperature data is not possible. Future studies will aim to include actual temperature measurements to provide a more comprehensive validation of the simulation results. Despite this limitation, the trends observed in the simulation provide valuable insights into the behavior of the molding compound under the conditions studied.

At P2, the calculated pressure is slightly overpredicted as the mold filling progresses toward the switchover time point. Also, at P3, the calculated pressure is substantially overpredicted across all given processing conditions and RTC compositions. This discrepancy arises due to the application of the no-slip boundary condition (where the fluid does not move relative to the wall) instead of the non-ideal wall slip (where fluid slides on the wall) at the wall. The impact of non-ideal wall slip on internal mold pressure development during the mold-filling phase is substantial, particularly in the processing of highly filled thermoset composites [13]. Consequently, the calculated pressure values are higher than those measured in the experiments. A similar observation was encountered while comparing the pressure profiles at the farthest pressure sensor along the flow path with the no-slip boundary condition in [12,14,16,37].

**Figure 10:**
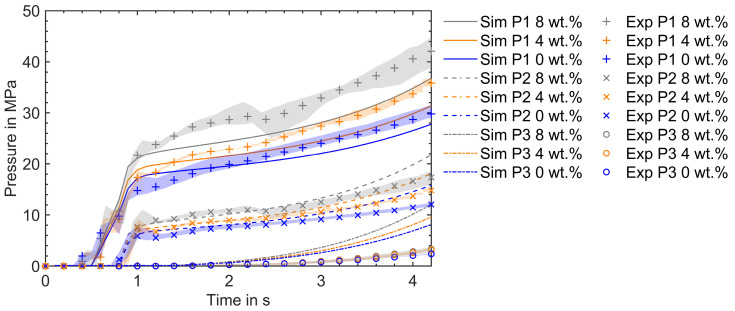
Pressure profiles during the filling phase at a flow rate of 15 cm^3^/s. The simulation and experimental results are plotted for different RTC proportions.

**Figure 11:**
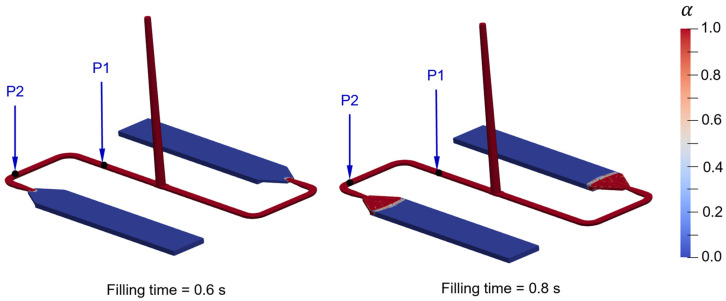
Melt front position during the mold filling time at which it enters and leaves the film gate. The mold-filling behavior corresponds to VTC reinforced with 8% RTC at an injection speed of 15 cm^3^/s.

Figure 10 illustrates the comparison of the results between 0 wt.%, 4 wt.%, and 8 wt.% recycled content at a flow rate of 15 cm^3^/s. The simulation results demonstrate qualitative agreement with the experimental outcomes, distinctly capturing the offset among the pressure profiles for different proportions of RTC. This indicates that the implemented viscosity model can reproduce the experimental outcomes for multiple flow rates. Nonetheless, discrepancies do emerge at probing points P1 and P2 because of the increased pressure required to force the polymer melt to flow through the film gates (thin sections). In the experimental trials involving the reinforcement of RTC fillers, there is an increase in pressure at the beginning of mold filling. This increase could be attributed to the formation of a plug at the flow front, which experiences more friction as the polymer melt flows through the flow channels and film gate. Once it exits the film gate, it loses contact with the wall, resulting in a decrease in pressure. The positions of the melt front at entering and leaving the film gate are shown in Figure 11.

**Figure 12:**
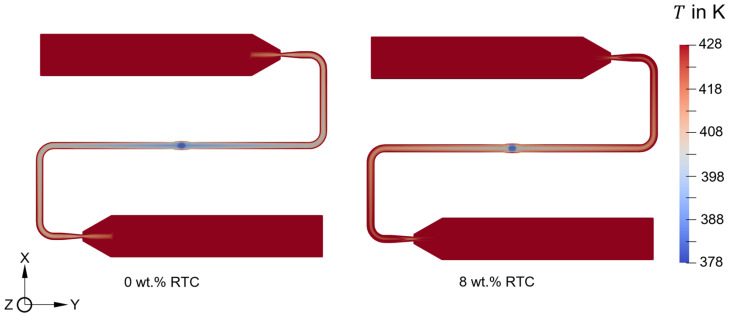
Comparison of temperature on an XY-plane at Z = 135.5 mm (Z = 0 mm corresponds to the Inlet boundary patch) at fill time = 1.4 s. The results are plotted for an injection speed of 15 cm^3^/s and a mold surface temperature of 175 °C.

The rise in viscosity resulting from an increase in filler content due to reinforcement of RTC significantly affects the shear heating of the polymer melt during mold filling (cf. Equation (5)). Figure 12 presents a comparison of the temperature field during the mold-filling process between 0 wt.% and 8 wt.% RTC at identical processing conditions. It is evident that the temperature of the polymer melt with 8 wt.% RTC content is higher than that with 0 wt.% RTC content in both the flow channel and film gate. This difference in temperature is attributed to the elevated shear heating observed in the polymer with a higher RTC content.

## 6. Conclusions

In this contribution, a mold-filling simulation approach for polydisperse suspensions of recycled and virgin thermoset composites (RTC and VTC) is presented. The mold-filling behavior in a reactive injection molding (RIM) process was simulated where the VTC was reinforced with different propositions of RTC. To account for the effect of reinforced RTC on the mold-filling behavior, the Cross–Castro–Macosko (CCM) model for the viscosity of homogeneous thermoset matrix–filler suspensions was phenomenologically extended using the stress–strain amplification approach presented by Domurath et al. [11] for thermoplastic melts. The constitutive model for viscosity is a function of the extent of reinforcing fillers, in addition to temperature, shear rate, and degree of cure. This material model is implemented in the CFD code OpenFOAM 6. Mold-filling simulations were carried out by extending the multiphase VoF solver compressibleInterFoam. Qualitative agreement was found between the simulation and experimental results for different proportions of reinforcing RTC and different injection speeds. This indicates that the implemented viscosity model can reproduce the experimental mold-filling behavior by varying the reinforcement of RTC content.

In this study, the viscosity model considers the impact of reinforcing spherical-shaped fillers. However, by assessing intrinsic viscosity as a function of aspect ratio, the model remains versatile, enabling the consideration of the reinforcement effects of anisotropic fillers with varying aspect ratios. This flexibility is advantageous, particularly during the compounding stage. When the viscosity of the neat resin is known, the viscosity can readily be determined for different proportions of reinforcing fillers. Nonetheless, the model lacks the influence of the instantaneous orientation of anisotropic fillers on the viscosity during the mold-filling phase.

Besides the influence of reinforcing fillers on viscosity, their influence on the shear heating and resulting temperature distribution during the mold-filling process is also considered and numerically demonstrated. Nevertheless, some discrepancies between simulation and experimental results are still observed. They can be attributed to different contributing factors, e.g., potential errors during data fitting in the rheological characterization and numerical errors in the simulations. Additionally, a consideration of the aforementioned wall-slip and mold–melt heat transfer will improve the accuracy of flow modeling. Since the OpenFOAM source code is accessible and modifiable, models for wall-slip and mold–melt heat transfer can be implemented.

## Figures and Tables

**Table 1:** Shape characteristics of the fillers and volume fractions.

Filler	$A_{r}$	Mean Diameter in μm	Fillers’ Volume Fractions		
			Composition 1	Composition 2	Composition 3
Glass fibers	15.38	13	0.140	0.134	0.128
Wollastonite	45	12.5	0.328	0.313	0.298
Silicon dioxide	1	40	0.225	0.214	0.204
RTC	1	127.27	0	0.046	0.092

**Table 2:** Values of variables in the initial internal field and boundary patches.

Variable	Unit	Initial Internal Field	Inlet	Wall	Outlets 1 and 2
α	-	0	fixedValue 1	zeroGradient	zeroGradient
T	K	448.15	fixedValue 378.15	fixedValue 448.15	zeroGradient
v	$m^{3}/s$	(0 0 0)	flowRateInletVelocity 10^−5^ or 1.5 × 10^−5^	zeroGradient	zeroGradient
p	Pa	10^5^	fixedfluxPressure 10^5^	fixedfluxPressure 10^5^	fixedfluxPressure 10^5^
$p_{rgh}$	Pa	10^5^	fixedfluxPressure 10^5^	fixedfluxPressure 10^5^	fixedfluxPressure 10^5^
c	-	0.001	zeroGradient	zeroGradient	zeroGradient
$\dot{c}$	1/s	0	zeroGradient	zeroGradient	zeroGradient

**Table 3:** Parameters of the reactive viscosity model corresponding to Equations (13), (14), and (25).

Parameter	Value	Unit
n	0.477	−
$\tau^{*}$	1.0595	Pa
B	1.5 × 10^−8^	Pa s
$T_{b}$	15,500	K
$c_{1}$	4	−
$c_{2}$	44	−
$c_{g}$	0.4	−

**Table 4:** Parameters of the curing kinetics model corresponding to Equations (26)–(28).

Parameter	Value	Unit
$R_{g}$	8.314	$J/\left(mol\;K\right)$
$A_{1}$	9.55 × 10^12^	1/s
$A_{2}$	0.0063	1/s
$E_{1}$	122,000	J/mol
$E_{2}$	18,872.9	J/mol
m	0.001	−
n	2.2	−

## Data Availability

The data presented in this study are available upon request from the corresponding author.
